# Impact of endometriosis on female sexual function: an updated systematic review and meta-analysis

**DOI:** 10.1093/sexmed/qfad026

**Published:** 2023-05-29

**Authors:** Xiujuan Zhu, Yanhui Wu, Jing Jia, Xinwei Zhao, Xiuping Zhao

**Affiliations:** Department of Gynecology, the Third Affiliated Hospital of Beijing University of Traditional Chinese Medicine, Chaoyang District, Beijing, China; Department of Gynecology, the Third Affiliated Hospital of Beijing University of Traditional Chinese Medicine, Chaoyang District, Beijing, China; Department of Gynecology, the Third Affiliated Hospital of Beijing University of Traditional Chinese Medicine, Chaoyang District, Beijing, China; Department of Reproductive Medicine, Cangzhou Hospital of Integrated Traditional and Western Medicine, Hebei Province, Beijing, China; Department of Gynecology, the Third Affiliated Hospital of Beijing University of Traditional Chinese Medicine, Chaoyang District, Beijing, China

**Keywords:** endometriosis, sexual function, female sexual function index, systematic review, meta-analysis

## Abstract

**Introduction:**

Endometriosis can lead to a state of chronic inflammation marked by the presence of scarring and adhesions within the pelvis and/or other parts of the body. Recent estimates suggest that globally this condition affects approximately 10% of women in the reproductive age group.

**Aims:**

In this study we sought updated evidence on the association between endometriosis and sexual function in female patients.

**Methods:**

We used standard assessment tools to conduct a systematic search of the PubMed, EMBASE, and Scopus databases for observational studies that documented the association of endometriosis with female sexual function. A random-effects model was used for the analysis, and effect sizes were reported as the weighted mean difference (WMD) or OR with 95% CIs.

**Results:**

A total of 13 studies were selected for inclusion in our investigation. All of the included studies were cross-sectional in design. The data on sexual function in most of the studies were collected by using the Female Sexual Function Index (FSFI) tool, for which higher scores suggest better sexual function. The risk of sexual dysfunction (based on specific cutoffs for the FSFI score) was higher in women with than in women without endometriosis (OR 1.71; 95% CI, 1.21-2.43). In addition, when we used continuous scores to examine the risk of sexual dysfunction, diagnosis of endometriosis was associated with significantly lower overall FSFI scores (WMD, −3.40; 95% CI, −5.13 to −1.66) and lower scores on all of its 6 domains, ie, desire (WMD, −0.27; 95% CI, −0.53 to −0.02), arousal (WMD, −0.43; 95% CI, −0.79 to −0.07), lubrication (WMD, −0.49; 95% CI, −0.66 to −0.31), orgasm (WMD, −0.65; 95% CI, −1.07 to −0.23), satisfaction (WMD, −0.52; 95% CI, −0.77 to −0.26), and pain (WMD, −1.06; 95% CI, −1.57 to −0.55).

**Conclusion:**

The findings of this study suggest that female patients with endometriosis have suboptimal sexual function compared with healthy female subjects. Patients with endometriosis should be offered sexual counseling and supportive care by a multidisciplinary team of gynecologists, psychologists, and sexual therapists.

## Introduction

Endometriosis is a clinical condition characterized by the presence of endometrium-like tissue outside the uterus.^1^^,^^2^ Common symptoms of endometriosis include menstrual irregularities, infertility, pelvic pain, dyspareunia, and dysmenorrhea.^1^^,^^2^ Endometriosis may lead to a state of chronic inflammation marked by the presence of scarring and adhesions within the pelvis and/or other parts of the body.^1^^,^^2^ Recent global estimates suggest that this condition affects an estimated 10% of women in the reproductive age group (approximately 170 million) worldwide.^3^

Studies have shown that endometriosis may affect patient psychological well-being, sexual functioning, and social relationships.^4^ The quality of sexual life is a very important element of overall quality of life.^5^ Patients with endometriosis often complain of dyspareunia, ie, painful sex, overall impaired sexual functioning, and decreased satisfaction,^6^^,^^7^ which may negatively affect personal relationships.^8^ However, there is still no consensus on the magnitude and direction of this association, and current evidence comes mainly from studies with small sample sizes that have often had controversial results.^9-13^ In the most recently published systematic review that addresses this issue, Shi et al. analyzed findings from 6 studies.^14^ The included studies had used the female sexual functioning index (FSFI), a 19-item tool for assessing sexual function, with higher scores reflecting better function.^15^ Shi et al found that in women with endometriosis, FSFI scores were significantly lower than those for apparently healthy women.^14^ In another review, Perez-Lopez et al included 4 studies and found that women with endometriosis had an estimated 2 times higher risk of sexual dysfunction (OR 2.38) than women without endometriosis.^16^ In our updated literature search, we identified additional studies that were also performed to examine the association of endometriosis with female sexual function but were not included in the reviews by Shi et al.^14^ or Perez-Lopez et al.^16^ The goal of the current meta-analysis was to update the previous reviews and provide the most contemporary evidence on the association of endometriosis with sexual function. With the meta-analysis we specifically aimed to answer the following question: “Is sexual function decreased in women with endometriosis compared to women without this condition?”

## Methods

### Study selection

We developed a strategy to systematically search 3 databases, PubMed, EMBASE, and Scopus, for studies with documentation of an association between endometriosis and female sexual function. The search strategy is shown in Table S1. English language studies published from database inception until January 15, 2023, were eligible for inclusion. We followed the standard PRISMA (Preferred Reporting Items for Systematic Reviews and Meta-Analyses) guidelines.^17^

In the studies selected for inclusion, standard tools were used to acquire data on sexual function, and sexual function was compared in women with and women without endometriosis. We excluded studies that did not have a comparison group, ie, those that provided only descriptive data on the epidemiology of sexual dysfunction in women with endometriosis. With respect to the study design, we included only studies with an observational design. We did not examine the effects of endometriosis treatment on sexual function and therefore excluded randomized controlled trials and quasi-experimental studies. Reviews, case reports, and case series were also excluded.

Using the strategy outlined above, we performed the search strategy in the 3 databases. The total number of studies identified were reviewed, and duplicate studies were removed. After these steps, 2 of the study investigators independently screened the studies for potential inclusion by reviewing the title and the abstract of each study and making further exclusions. After these steps were completed, the full texts of the remaining studies were read, and decisions were made for final inclusion. Resolution of any discrepancies in this process was achieved through discussion with a senior author.

### Data extraction and statistical analysis

Data extraction was done by using a pretested electronic sheet consisting of variables such as study identifiers (name of first author, country where the study was conducted, and year of publication), study design, methodology for data collection on exposure and outcome(s), baseline characteristics of the study subjects, sample size, and important findings. The pooled effect size was reported as the weighted mean difference (WMD), along with the 95% CIs for continuous outcomes and ORs for categorical outcomes.

Random-effects models were used for all analyses to account for the variability in the included studies with respect to subject characteristics, study settings, sample sizes, and methods of data collection on exposure. These differences would have led to substantial heterogeneity in the reported findings. Risk of bias was assessed by using the Newcastle-Ottawa Scale.^18^ We used Egger’s test along with funnel-plot visual inspection to assess publication bias.^19^  *P* values < 0.05 were considered statistically significant. Post hoc subgroup and sensitivity analyses were conducted based on the study setting (upper-middle and lower-middle income and high-income settings, assessed per the World Bank Classification of economies),^20^ study participants (stage 3 or 4 endometriosis patients only), and pooling of findings from studies with a sample size of more than 100.

To test the robustness of our findings, we performed a sensitivity analysis that excluded the data from the study conducted by Kling et al.^25^ The rationale for the exclusion of this study was that the study population included only women who were comparatively older than the women in the other study populations. The control subjects in the Kling et al. study were recruited from women’s clinics and may have had other underlying symptoms and pathologies. Additionally, the reported sexual dysfunction rate in the control group of the Kling et al. study was notably higher than the rates in the other studies, and the number of controls was approximately 10 times higher than the number of endometriosis cases. All analysis was conducted using Stata version 16 (StataCorp LLC, College Station, TX).

### Ethical clearance

Since this meta-analysis drew upon previously published studies and did not involve direct human participation, ethical clearance was not required.

## Results

**Figure 1:**
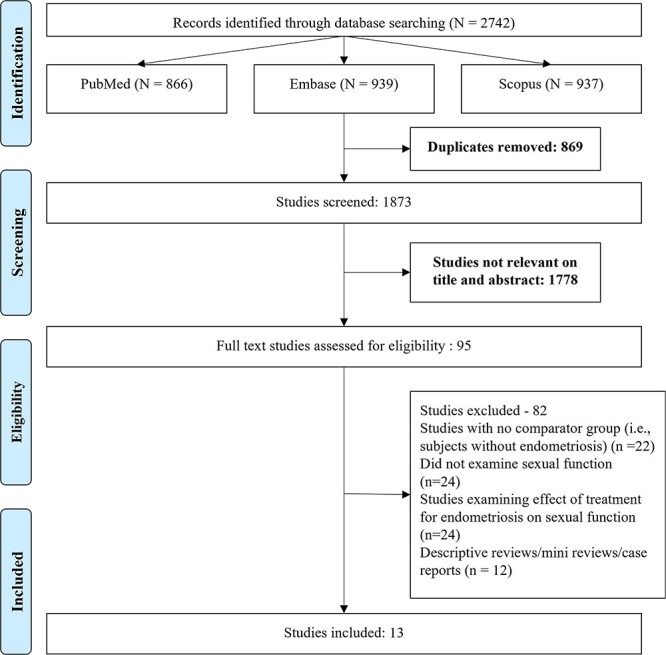
Selection process of studies included in the review.

We identified 2742 studies with the systematic literature search across the 3 databases. After removal of the 869 duplicates, 1873 unique studies remained. Title and abstract screening led to further exclusion of 1778 studies. The full texts of the remaining 95 studies were reviewed, and 82 studies were further excluded. The reasons for exclusion are summarized in Figure 1. Finally, 13 studies were chosen for inclusion in this meta-analysis.^9-13^^,^^21-28^

### Characteristics of the included studies

Table 1 presents the specific details of the included studies. All included studies were cross-sectional in design except 1 study that was a prospective case-control study. The majority of studies were conducted in Italy (*n* = 5), followed by Iran (*n* = 3) and Brazil (*n* = 2). One study each was conducted in China, the Netherlands, and the United States. Three studies each were conducted in low-middle and upper-middle income settings, respectively, and 7 studies were conducted in high-income settings. In all of the studies, except 1 study for which the data on endometriosis were self-reported, the diagnosis of endometriosis was made by using a standard gynecological examination and/or histopathological and imaging procedures (transvaginal ultrasonography or MRI). In 7 studies, the patients had stage 3 or 4 endometriosis. The remaining studies did not provide data on the stage of endometriosis of the study patients.

**Table 1:** Characteristics of the studies included in the meta-analysis.

**Author (year of publication)**	**Study design (country)**	**Sample size**	**Exposure and outcome assessment**	**Study participant characteristics**	**Total and domain FSFI scores for women with endometriosis vs healthy controls, mean (SD)**	**Risk of sexual dysfunction, total FSFI or GSSI score (OR [95% CI])**
Daneshfar et al (2022)^21^	Cross-sectionalwith convenience sampling (Iran)	420 (210 controls, 210 endometriosis patients)	Endometriosis: laparoscopy confirmed Sexual function: FSFI tool	Mean age approximately 31 years; mean BMI approximately 26 Endometriosis group: primary infertility in 85%, stage 3 or 4 endometriosis in 90% Control group: more educated No baseline difference between groups in depression and anxiety scores *Characteristics of controls:* healthy women of reproductive age without history of reproductive problems; selected from women referred to study clinic	Total, 23.44 (2.6) vs 28.5 (2.5) Desire, 4.1 (0.96) vs 4.3 (1.1) Arousal, 4.6 (1.3) vs 4.8 (1.5) Lubrication, 4.3 (0.7) vs 5.0 (0.9) Orgasm, 3.0 (1.0) vs 4.7 (0.8) Satisfaction, 3.7 (1.0) vs 4.7 (0.8) Pain, 3.5 (0.9) vs 4.5 (0.9)	
Rossi et al (2022)^10^	Cross-sectional with snowball sampling (Italy)	187 (100 controls, 87 endometriosis patients)	Endometriosis: gynecological examination and transvaginal ultrasonography Sexual function: FSFI tool	Mean age 35 years; Women on oral contraceptives or progestogen eligible, those on gonadotropin-releasing hormone analogue excluded, sexual problem in past 6 months reported in more patients with endometriosis (51%) than subjects without (19%) *Characteristics of controls:* women with no reported reproductive health problems from general population, selected with web-based survey	Total, 22.49 (9.66) vs 24.81 (10.14) Desire, 3.79 (1.11) vs 3.86 (1.19) Arousal, 3.76 (1.82) vs 3.97 (2.01) Lubrication, 4.01 (2.18) vs 4.52 (2.2) Orgasm, 3.62 (2.19) vs 3.96 (2.21) Satisfaction, 4.06 (1.66) vs 4.09 (1.66) Pain, 3.26 (2.12) vs 4.42 (2.33)	
Yang et al (2021)^12^	Prospective unmatched case-control (China)	140 (63 controls, 77 endometriosis patients)	Endometriosis: laparoscopy followed by pathological examination. Sexual function: FSFI tool	Most subjects aged 20-40 years (80%), no documented psychological morbidity or chronic disease, no recent hormonal therapy, no baseline difference among those with vs without endometriosis All patients with endometriosis had stage 3 or 4 *Characteristics of controls:* women selected from study hospital, no reproductive health problems, no hormonal therapy in past 3 months	Total, 25.5 (1.1) vs 26.7 (0.85) Desire, 3.0 (0.2) vs 3.0 (0.19) Arousal, 3.6 (0.25) vs 3.6 (0.2) Lubrication, 4.8 (0.2) vs 5.1 (0.2) Orgasm, 4.0 (0.33) vs 4.4 (0.13) Satisfaction, 4.8 (0.22) vs 5.2 (0.14) Pain, 4.8 (0.37) vs 5.2 (0.27)	
Evangelista et al (2014)^9^	Cross-sectional (Brazil)	95 (38 controls, 57 endometriosis patients)	Endometriosis: clinical examination and transvaginal USG or MRI or histopathological examination Sexual function: FSFI tool	Mean age approximately 35 years, BMI approximately 25, no baseline difference (age/BMI/parity) in subjects with vs without endometriosis All patients with endometriosis had stage 3 or 4 *Characteristics of controls:* age 18-45 years, sexually active, no severe dysmenorrhea, no clinical or surgical evidence of endometriosis, normal gynecological examination	Total, 23.4 (8.7) vs 24.3 (6.5) Desire, 3.2 (1.2) vs 3.3 (1.2) Arousal, 3.7 (1.6) vs 3.8 (1.2) Lubrication, 4.3 (1.7) vs 4.2 (1.4) Orgasm, 4.2 (1.8) vs 4.1 (1.4) Satisfaction, 4.3 (1.7) vs 4.3 (1.5) Pain, 3.4 (1.8) vs 4.5 (1.7)	≤26.55 (1.53 [0.65-3.64])
De Graaff et al (2016)^13^	Cross-sectional (Netherlands)	123 (40 control, 83 endometriosis patients)	Endometriosis: laparoscopy or open surgery, pelvic examination/USG/MRI Sexual function: FSFI tool	Mean age approximately 34 years, higher parity in those without endometriosis, higher proportion of fertility treatment in those with endometriosis, similar proportion of psychiatrist/psychologist visits in both groups All patients with endometriosis had stage 3 or 4 *Characteristics of controls:* Women visiting outpatient department for contraception-related issues (oral, intrauterine device, request for sterilization)	Total 25.4 (1.93) vs 30.6 (1.0) Desire, 3.0 (0.2) vs 3.6 (0.2) Arousal, 4.5 (0.4) vs 5.3 (0.3) Lubrication, 5.1 (0.4) vs 5.7 (0.1) Orgasm, 4.8 (0.5) vs 5.2 (0.2) Satisfaction, 4.8 (0.4) vs 5.2 (0.2) Pain, 4.0 (0.67) vs 6.0 (0.13) Risk of sexual dysfunction	
Mahsa et al (2014)^22^	Cross-sectional (Iran)	126 (80 controls, 46 endometriosis patients)	Endometriosis: laparoscopy confirmed Sexual function: FSFI tool	Mean age approximately 33 years, higher BMI in endometriosis patients (mean 26) *Characteristics of controls:* healthy subjects selected from patient’s families and/or hospital staff	Total, 20.5 (7.7) vs 24.3 (7.0) Desire, 3.3 (1.1) vs 3.6 (1.3) Arousal, 2.9 (1.4) vs 3.8 (1.3) Lubrication, 3.8 (1.5) vs 4.3 (1.4) Orgasm, 3.4 (1.6) vs 4.0 (1.5) Satisfaction, 3.8 (1.8) vs 4.3 (1.2) Pain, 3.4 (1.9) vs 4.2 (1.5)	<26 (2.02 [0.57-7.21])
Melis et al (2015)^23^	Cross-sectional (Italy)	81 (40 controls, 41endometriosis patients)	Endometriosis: clinical history and/or MRI/USG/surgically confirmed Sexual function: FSFI tool	Age range 18-45 (mean 31) years and mean BMI 21, no history of hormonal treatment or treatment for depression All patients with endometriosis had stage 3 or 4 *Characteristics of controls:* Volunteers consecutively recruited among women coming to study clinic for screening for cervical pathology without history of chronic pain, endometriosis, or other gynecological diseases	Total, 22.53 (2.0) vs 25.65 (2.3) Desire, 3.54 (1.5) vs 4.16 (1.17) Arousal, 3.72 (2.0) vs 4.04 (2.16) Lubrication, 4.11 (2.18) vs 4.3 (2.33) Orgasm, 3.97 (2.2) vs 4.29 (2.37) Satisfaction, 4.08 (2.03) vs 4.32 (2.05) Pain, 3.11 (1.93) vs 4.54 (2.32)	≤ 26.55 (1.75 ([0.72-4.24])
Somigliana et al (2020)^11^	Cross-sectional (Italy)	292 (230 controls, 62 patients with endometriosis)	Endometriosis: USG or presence of deep invasive lesions at time of in vitro fertilization Sexual function: FSFI tool	Mean age 38 years, mean BMI 22, no history of depression treatment, baseline characteristics similar in both groups All patients with endometriosis had stage 3 or 4 *Characteristics of control* Women with infertility and attending the IVF clinic; no history of chronic pain or endometriosis or other evident gynecological diseases	Total, 26.2 (7.5) vs 27.4 (6.8) Desire, 3.8 (1.1) vs 3.8 (1.1) Arousal, 4.3 (1.5) vs 4.4 (1.4) Lubrication, 4.6 (1.6) vs 4.9 (1.4) Orgasm, 4.2 (1.6) vs 4.5 (1.5) Satisfaction, 4.6 (1.4) vs 4.8 (1.2) Pain, 4.6 (1.6) vs 4.9 (1.4)	<26.5 (1.42 [0.80-2.54])
Ashrafi et al (2022)^24^	Cross-sectional (Iran)	240 (160 controls, 80 endometriosis patients)	Endometriosis: based on laparoscopy findings Sexual function: FSFI tool	Mean age 32 years, BMI 26, baseline proportion with anxiety and/or depression similar in both groups *Characteristics of control:* Fertile women recruited from healthcare centers, all had used a condom as a birth control method	Total 22.46 (1.84) vs 29.07 (2.50) Desire, 3.78 (0.70) vs 4.39 (1.14) Arousal, 3.88 (1.07) vs 5.03 (1.05) Lubrication, 4.29 (0.61) vs 4.99 (0.83) Orgasm, 3.00 (0.77) vs 4.49 (0.81) Satisfaction, 3.81 (0.73) vs 5.10 (0.78) Pain, 3.68 (0.74) vs 5.05 (0.85)	
Kling et al (2022)^25^	Cross-sectional (USA)	1040 (978 controls and 62 endometriosis patients)	Endometriosis: self-reported Sexual function: FSFI tool	Demographic data in the study presented for the overall sample; for this analysis, only premenopausal women were included. In overall sample, mean age approximately 50 years, 22% obese, 90% of White race, menopausal (60%), smoking (approximately 24%) *Characteristics of controls* Based on self-reporting of nondiagnosis of endometriosis, possibility that underlying undetected endometriosis is present, or some underlying symptoms/pathology; rate of sexual dysfunction very high (approximately 70%)		≤26.55 (2.30 [1.13-4.68])
Tripoli et al (2011)^26^	Cross-sectional (Brazil)	134 (50 controls, 84 endometriosis patience)	Endometriosis: laparoscopy followed by pathological examination Sexual function: GRISS	Age range 18-50 years, similar age, educational, and marital status among those with and without endometriosis *Characteristics of controls:* healthy patients with no gynecological disease attending the study clinic; none had history of dysmenorrhea, dyspareunia, or infertility	Significant reductions in sexual frequency and satisfaction; significant increase in sexual aversion, lack of expression of sensuality and vaginismus in those with endometriosis compared to healthy controls	
Ferrero et al (2005)^27^	Cross-sectional (Italy)	136 (40 control s, 96 endometriosis patients)	Endometriosis: laparoscopy followed by pathological examination. Sexual function: GSSI and sexual satisfaction subscale of the Derogatis Sexual Functioning Inventory	Mean age approximately 34 years, mean age at first intercourse approximately 20 years; all patients with endometriosis had stage 3 or 4 *Characteristics of controls:* Never surgically treated for endometriosis, no detectable endometriotic lesions at the time of surgical examination, did not use oral contraceptives or hormonal agents in previous 6 months	Total GSSI score, 3.3 (1.5) vs 4.8 (1.0) Desire, 2.6 (1.2) vs 2.5 (1.2) Orgasm, 2.3 (1.0) vs 3.4 (1.5) Satisfaction, 2.7 (1.5) vs 3.7 (1.8) Pain, 2.8 (1.0) vs 4.1 (1.3)	
Giuliani et al (2016)^28^	Cross-sectional (Italy)	300 (150- control and 150- with endometriosis)	Endometriosis: gynecological examination, transvaginal ultrasound, and MRI Sexual function: MFSQ	Mean age approximately 36 (range 22-50) years, higher proportion of those with endometriosis had no children. *Characteristics of controls:* volunteers, healthy women, aged 23-50 years, matched for age and relationship status	Those with endometriosis had significantly lower sexual function scores, compared to controls	

BMI, body mass index in kg/m2; FSFI, female sexual function index; GRISS, Golombok

Rust Inventory of Sexual Satisfaction; GSSI, Global Sexual Satisfaction Index; MFSQ, McCoy Female Sexuality Questionnaire; MRI, magnetic resonance imaging; USG, ultrasonography.

The data on sexual function in the majority of the studies (*n* = 10) were collected using the FSFI tool. FSFI is a self-reported tool that assesses sexual function over the preceding 4 weeks.^15^ The FSFI contains 19 items and collects data on 6 domains of sexual function: desire, arousal, vaginal lubrication, orgasm, satisfaction, and pain. For each domain except the pain domain, the item scores range from 0 to 5. Higher item scores indicate better function. Items in the pain domain are coded by a descending scale. To obtain the total FSFI score, the item scores within each domain are added and then multiplied by a correction factor. The resulting scores within each of the 6 domains are added to obtain a total FSFI score. Higher scores reflect better sexual function.^15^ In the remaining 3 studies, other tools such as the Golombok Rust Inventory of Sexual Satisfaction (GRISS), Global Sexual Satisfaction Index (GSSI), Sexual Satisfaction Subscale of the Derogatis Sexual Functioning Inventory, and the McCoy Female Sexuality Questionnaire (MFSQ) were used to collect data on sexual function.^29-31^ The quality assessment is presented in Table S2 and Table S3. All studies were of good quality, with scores ranging from 7 to 9 out of the maximum attainable score of 9.

### Findings from pooled analysis

**Figure 2:**
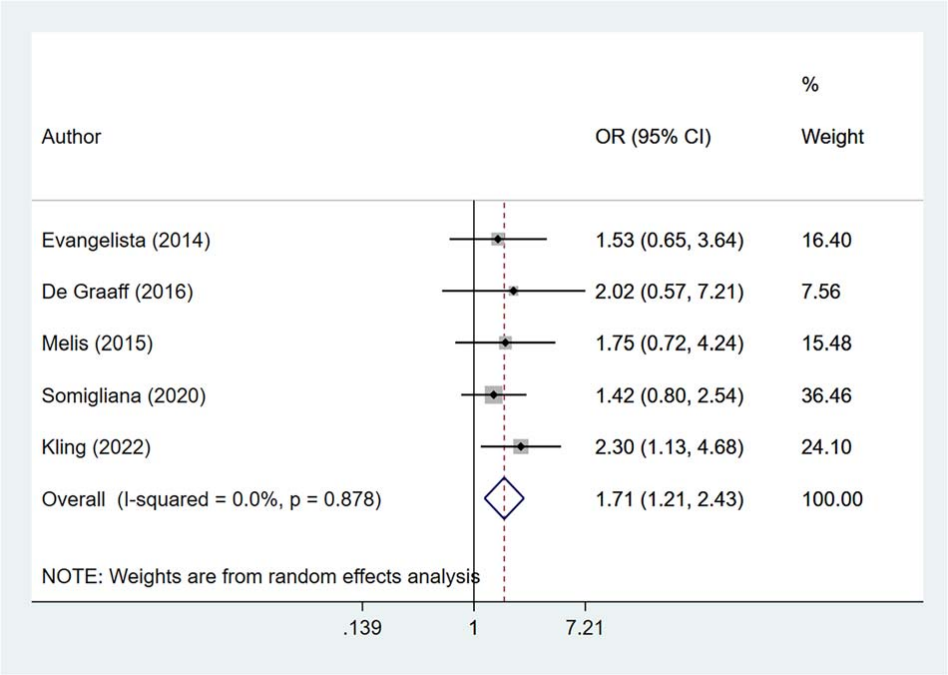
Pooled risk of sexual dysfunction in patients with and without endometriosis.

**Figure 3:**
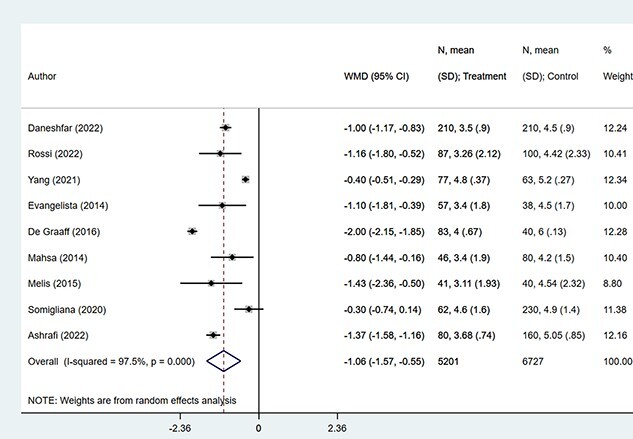
Sensitivity analysis for the pooled risk of sexual dysfunction in patients with and without endometriosis, after excluding the study by Kling et al.^25^

**Figure 4:**
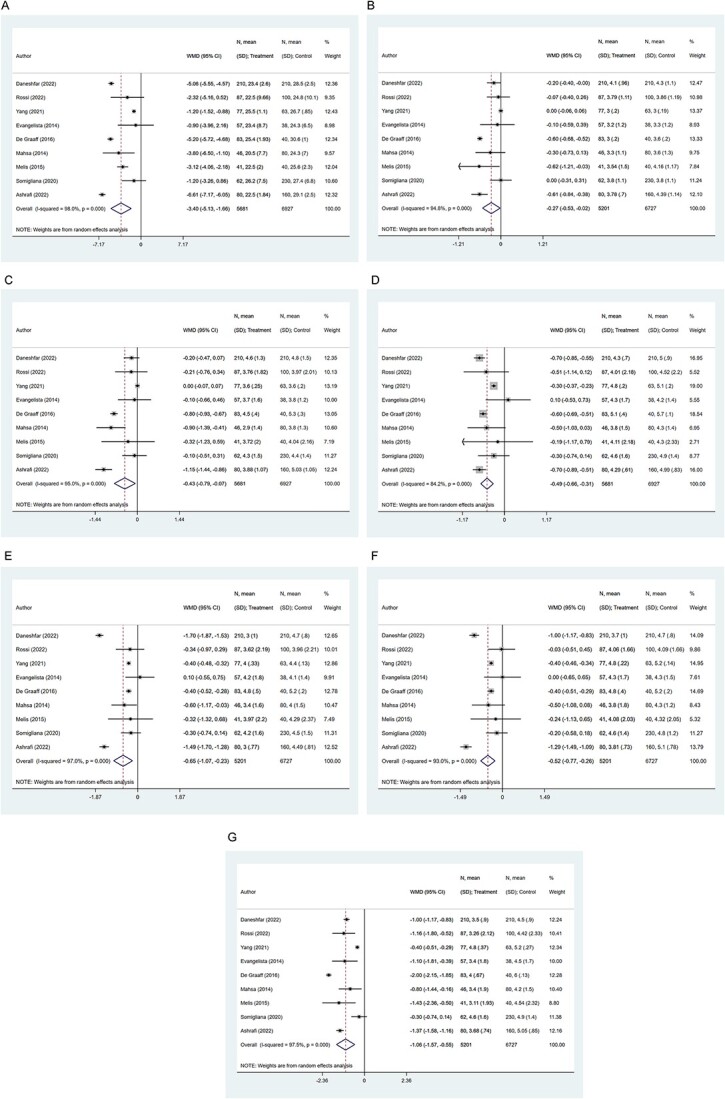
Comparison of mean scores between patients with and without endometriosis according to the total FSFI score (A) and scores in the 6 domains: desire (B), arousal (C) lubrication (D), orgasm (E), satisfaction (F), and pain (G). FSFI, Female Sexual Dysfunction Index.

The overall risk of sexual dysfunction (based on specific cutoffs for the FSFI score) was increased in patients with endometriosis (OR, 1.71; 95% CI, 1.21-2.43; *n* = 5, *I*^2^ = 0.0%), compared to those without endometriosis (Figure 2). Upon exclusion of the study by Kling et al,^25^ the pooled estimates still indicated an increased risk of sexual dysfunction in those with endometriosis (OR, 1.56; 95% CI, 1.05-2.33; *n* = 4, *I*^2^ = 0.0%) (Figure 3). Women with endometriosis had significantly lower scores for the total FSFI (WMD, −3.40; 95% CI, −5.13 to −1.66; *n* = 9, *I*^2^ = 98.0%) and the domains of desire (WMD, −0.27; 95% CI, −0.53 to 0.02; *n* = 9, *I*^2^ = 94.8%), arousal (WMD, −0.43; 95% CI, −0.79 to −0.07; *n* = 9, *I*^2^ = 95.0%), lubrication (WMD, −0.49; 95% CI, −0.66 to −0.31; *n* = 9, *I*^2^ = 84.2%), orgasm (WMD, −0.65; 95% CI, −1.07 to −0.23; *n* = 9, *I*^2^ = 97.0%), satisfaction (WMD, −0.52; 95% CI, −0.77 to −0.26; *n* = 9, *I*^2^ = 93.0%), and pain (WMD, −1.06; 95% CI, −1.57 to −0.55; *n* = 9, *I*^2^ = 97.5%) (Figure 4A-G). Egger’s test did not detect any evidence of publication bias for the outcomes considered (*P* > 0.05). Similarly, inspection of the funnel plots showed the lack of publication bias (Figures S1-S7).

Studies utilizing tools other than FSFI have also reported lower sexual function in women with than women without endometriosis (Table 1).

Sensitivity analysis of the studies in which most of the women participants were diagnosed with stage 3 or 4 endometriosis showed that endometriosis was associated with lower total FSFI scores (WMD, −2.93; 95% CI, −4.87 to −0.98; *n* = 6, *I*^2^ = 98.1%) and scores for the domains of lubrication (WMD, −0.43; 95% CI, −0.65 to −0.22; *n* = 6, *I*^2^ = 88.4%), orgasm (WMD, −0.55; 95% CI, −1.04 to −0.05; *n* = 6, *I*^2^ = 97.4%), satisfaction (WMD, −0.46; 95% CI, −0.69 to −0.23; *n* = 6, *I*^2^ = 89.1%), and pain (WMD, −1.03; 95% CI, −1.72 to −0.33; *n* = 6, *I*^2^ = 98.3%) but not the domains of desire and arousal (Table 2). Study results from both low- and upper-middle income as well as high-income settings suggest that women with endometriosis have lower total FSFI scores and lower scores for the domains of lubrication, orgasm, satisfaction, and pain than women without endometriosis. Similar findings suggestive of comparatively poorer sexual function in patients with endometriosis were found when studies with a sample size of more than 100 were pooled together (Table 2).

**Table 2:** Findings of the subgroup and sensitivity analysis.

	**FSFI score, WMD (95% CI)**						
	**Total**	**Desire**	**Arousal**	**Lubrication**	**Orgasm**	**Satisfaction**	**Pain**
Low or upper-middle income	−3.62 (−6.32 to −0.92) [N = 5; *I*^2^ = 98.9%]	−0.24 (−0.50 to 0.02) [N = 5; *I*^2^ = 85.6%]	−0.46 (−0.96 to 0.03) [N = 5; *I*^2^ = 94.2%]	−0.48 (−0.74 to −0.22) [N = 5; *I*^2^ = 88.8%]	−0.85 (−1.57 to −0.13) [N = 5; *I*^2^ = 98.3%]	−0.68 (−1.14 to −0.23) [N = 5; *I*^2^ = 96.2%]	−0.93 (−1.39 to −0.46) [N = 5; *I*^2^ = 95.2%]
High income	−3.18 (−5.01 to −1.36) [N = 4; *I*^2^ = 89.0%]	−0.31 (−0.69 to 0.06) [N = 4; *I*^2^ = 86.4%]	−0.40 (−0.86 to 0.06) [N = 4; *I*^2^ = 78.8%]	−0.58 (−0.67 to −0.49) [N = 4; *I*^2^ = 0.0%]	−0.39 (−0.51 to −0.27) [N = 4; *I*^2^ = 0.0%]	−0.36 (−0.47 to −0.24) [N = 4; *I*^2^ = 3.3%]	−1.22 (−2.20 to −0.25) [N = 4; *I*^2^ = 94.7%]
**Stage 3 or 4 endometriosis**	−2.93 (−4.87 to −0.98) [N = 6; *I*^2^ = 98.1%]	−0.24 (−0.57 to 0.08) [N = 6; *I*^2^ = 96.5%]	−0.26 (−0.67 to 0.15) [N = 6; *I*^2^ = 95.6%]	−0.43 (−0.65 to −0.22) [N = 6; *I*^2^ = 88.4%]	−0.55 (−1.04 to −0.05) [N = 6; *I*^2^ = 97.4%]	−0.46 (−0.69 to −0.23) [N = 6; *I*^2^ = 89.1%]	−1.03 (−1.72 to −0.33) [N = 6; *I*^2^ = 98.3%]
**Sample size >100**	−3.71 (−5.75 to −1.68) [N = 7; *I*^2^ = 98.5%]	−0.26 (−0.54 to 0.02) [N = 7; *I*^2^ = 96.1%]	−0.48 (−0.88 to −0.08) [N = 7; *I*^2^ = 96.3%]	−0.53 (−0.71 to −0.35) [N = 7; *I*^2^ = 87.3%]	−0.77 (−1.23 to −0.30) [N = 7; *I*^2^ = 97.7%]	−0.58 (−0.85 to −0.30) [N = 7; *I*^2^ = 94.6%]	−1.01 (−1.59 to −0.44) [N = 7; *I*^2^ = 98.1%]

## Discussion

In the current review, we found that female patients with endometriosis had lower sexual function scores than those without endometriosis. The aim of this analysis was to update the evidence base through inclusion of data from more recently published studies on the association of endometriosis and sexual dysfunction. Our findings are similar to those of previous recent reviews on this subject.^8^^,^^14^^,^^32^ Our review included more studies (*n* = 13) than the previous 2 most recent reviews by Shi et al (*n* = 6 studies) and Perez-Lopez et al (*n* = 4 studies).^14^^,^^16^ We included all of the studies that were also included in these reviews. The reason for the additional studies in our review was mainly because our literature review was performed the most recently. Perez-Lopez et al searched databases until March 2020, while Shi et al reported the last date of their literature search as August 2021. However, there were 3 studies that should have been included in previous reviews but were not.^25-27^

Endometriosis is a clinical condition that is characterized by the presence of endometrial tissue outside of the uterine cavity, ie, in the pelvis, ovaries, etc.^1^^,^^3^ There is a marked increase in estrogen levels and consequent progesterone resistance in patients with endometriosis.^33^ The disease is characterized by the presence of chronic inflammation leading to pelvic pain and a decrease in endometrial receptivity for embryo implantation.^33^^,^^34^ All of these processes may lead to sexual dysfunction in females. One of the commonly suggested reasons for suboptimal sexual function is the associated dyspareunia and deep pelvic pain.^35^ Additionally, patients with endometriosis who are experiencing dyspareunia often report a sense of guilt toward their partner and a feeling of compromised femininity which in turn aggravates sexual dysfunction.^36^ Although the causal relationship has not been established, it is hypothesized that endometriosis is associated with mood disturbances, such as anxiety or depression, which may impair sexual function.^37^

Previous reviews that reported the link between endometriosis and sexual dysfunction were based on studies that used FSFI to evaluate sexual function.^15^^,^^16^ Our review and meta-analysis expanded these analyses by including studies utilizing tools other than the FSFI. Tripoli et al used the GRISS tool and documented significant reductions in sexual frequency and satisfaction, significant increases in sexual aversion, and lack of expression of sensuality and vaginismus in patients with endometriosis.^26^ Giuliani et al used the MFSQ and found that women with endometriosis had significantly lower sexual function scores than the control group.^28^ Taken together, our analysis consistently demonstrates that endometriosis is associated with decreased sexual function in women regardless of the method of assessment.

The results of our study further highlight the importance of counseling for patients with endometriosis. Women with this condition should be encouraged to communicate their sexual problems to the treating gynecologist, and a detailed sexual history should be a part of the comprehensive assessment protocol. A multidisciplinary team consisting of gynecologists, psychologists, and sexual therapists may be required for attainment of a favorable sexual function. Careful selection of the treatment modality for endometriosis is essential. Currently, the treatment of endometriosis is largely either hormonal therapy with the intent to attain a hypoestrogenic state or surgical removal of visible lesions.^38^ Both these management modalities have their respective side effects. Hormonal therapies are associated with varied adverse effects (such as mood changes and vaginal dryness that could negatively impact sexual function) and high risk of recurrence, while surgical management is associated with peri- and postoperative complications.^38^

The strength of the present review is the comprehensive inclusion of all available studies that have addressed the link of endometriosis with sexual dysfunction. Standard methodology for conducting the meta-analysis was followed. However, there are a few limitations that should be considered while interpreting the findings. First, the included studies were cross-sectional in design, and therefore causality cannot be conclusively established. Second, there was significant heterogeneity for most of the outcomes. We conducted subgroup and sensitivity analyses to elucidate the reasons for this high heterogeneity. However, as in most of the studies, the baseline characteristics of the patients were similar (with respect to subject age, BMI, parity, or history of mood disorders/anxiety/depression), and we were unable to further explore the reasons for heterogeneity. Third, for the outcome “risk of sexual dysfunction” (as a categorial variable), different studies used varied cutoffs, and this may have contributed to heterogeneity.

## Conclusion

The findings of our analysis suggest an association between the presence of endometriosis and female sexual dysfunction. This finding is important and has profound clinical implications. Female patients with this condition need to be provided with counseling and supportive therapy to alleviate their insecure mental status and enhance their self-efficacy. Evidence-based treatment for endometriosis with the intent to improve sexual function should be a part of the process of patient care management. There is also a need for comprehensive studies of the effects of suboptimal sexual function in women with endometriosis on the sexual and mental health of their spouses.

### Funding

The Third Affiliated Hospital of Beijing University of Traditional Chinese Medicine and the Horizontal Fund Project of Beijing University of Information Technology.

*Conflicts of interest*: The authors declare no conflict of interest.

## Data availability

The review protocol underlying this article is available at PROSPERO and can be accessed with the registration number CRD42023390667.

## Supplementary Material

Supplementary_figure_1_qfad026

Supplementary_figure_2_qfad026

Supplementary_figure_3_qfad026

Supplementary_figure_4_qfad026

Supplementary_figure_5_qfad026

Supplementary_figure_6_qfad026

Supplementary_figure_7_qfad026

Supplementary_table_1_qfad026

Supplementary_table_2_qfad026

Supplementary_table_3_qfad026
