# Supplementary figures and images for: Impact of endometriosis on female sexual function: an updated systematic review and meta-analysis
Source: Sex Med. 2023 May 29;11(2):qfad026. doi: 10.1093/sexmed/qfad026 (PMC10226816; doi:10.1093/sexmed/qfad026)

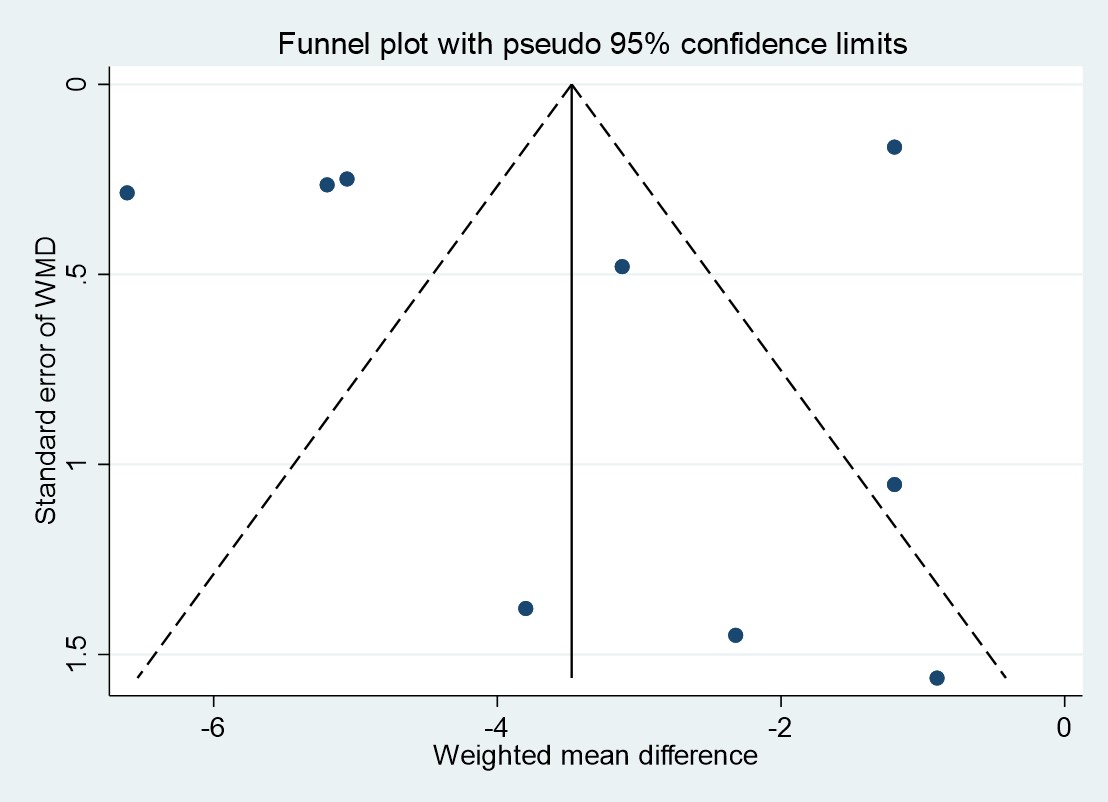

Supplement: Supplementary_figure_1_qfad026 [file supplementary_figure_1_qfad026.jpeg]

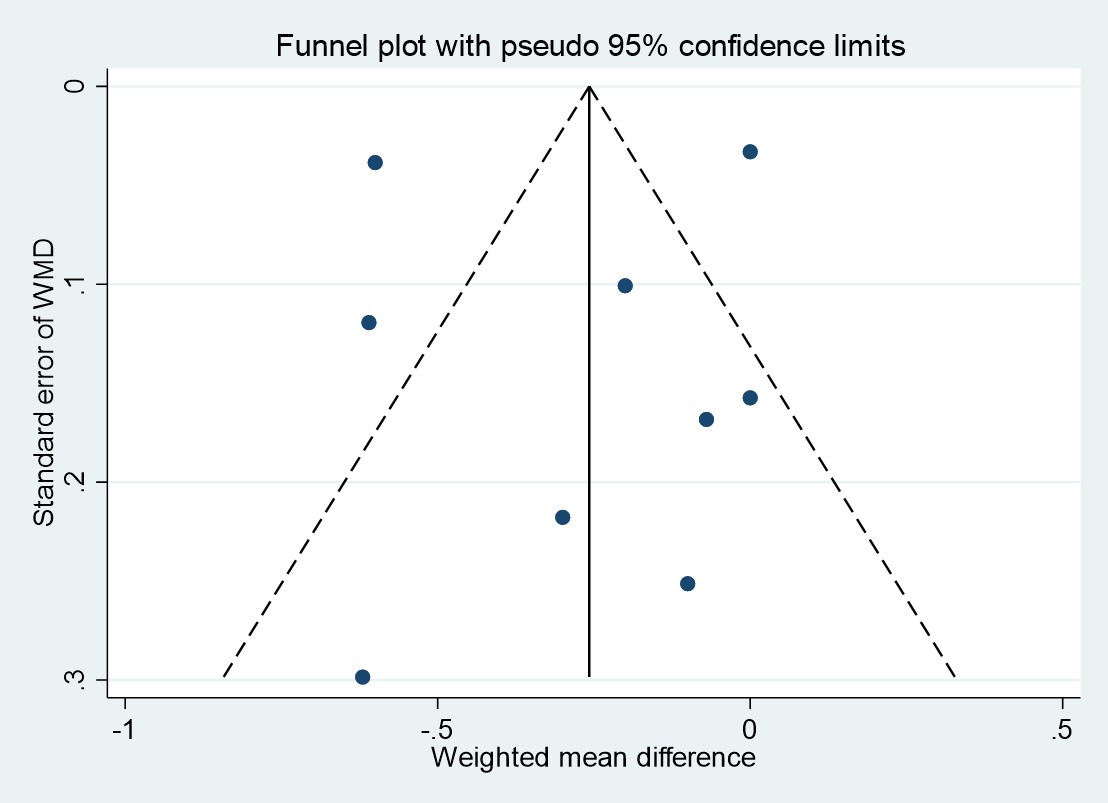

Supplement: Supplementary_figure_2_qfad026 [file supplementary_figure_2_qfad026.jpeg]

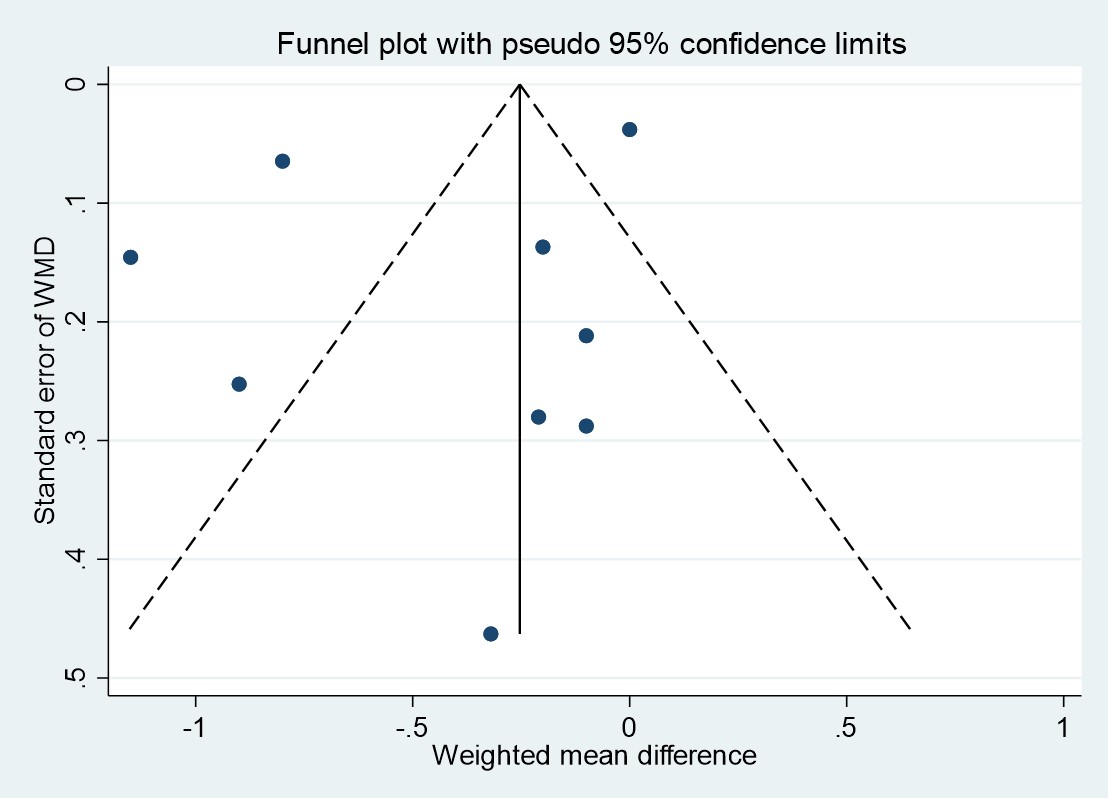

Supplement: Supplementary_figure_3_qfad026 [file supplementary_figure_3_qfad026.jpeg]

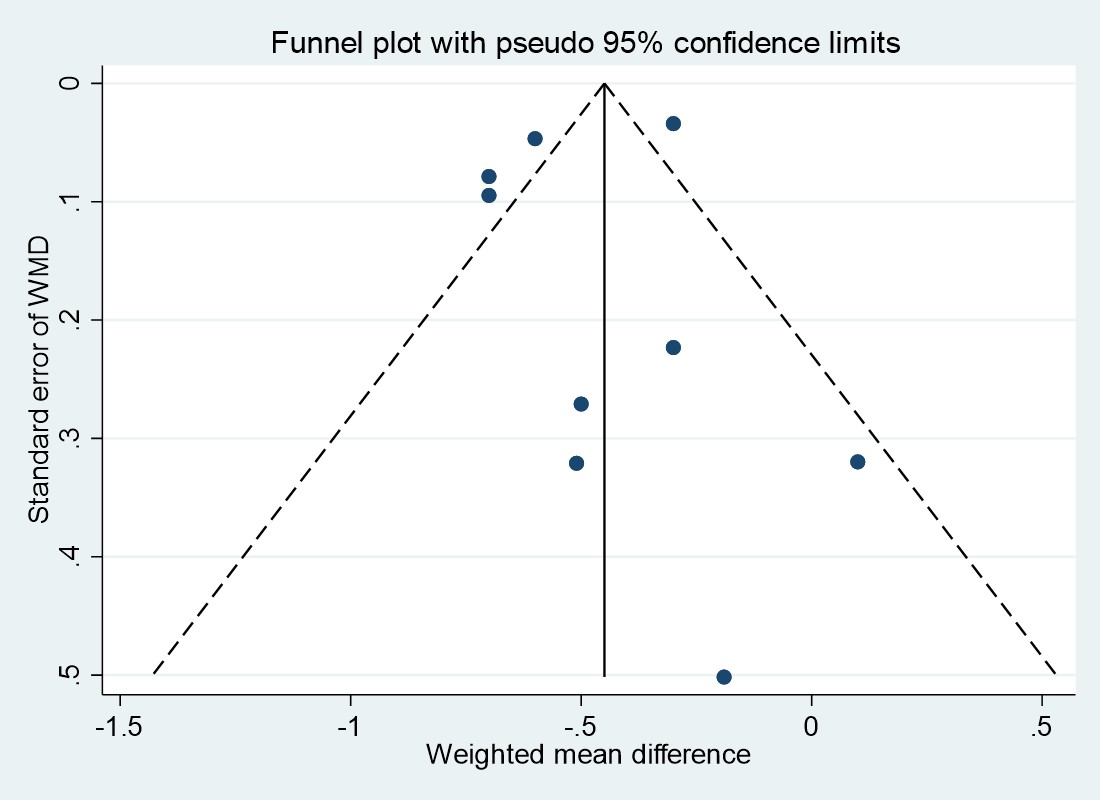

Supplement: Supplementary_figure_4_qfad026 [file supplementary_figure_4_qfad026.jpeg]

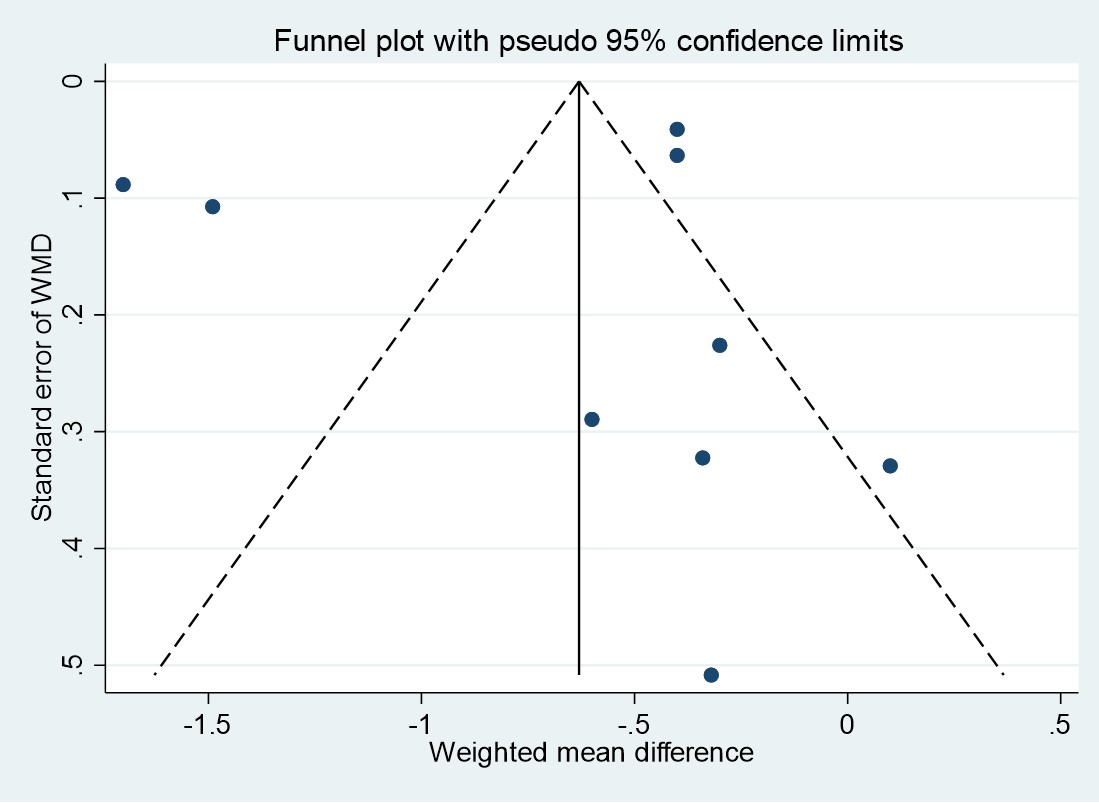

Supplement: Supplementary_figure_5_qfad026 [file supplementary_figure_5_qfad026.jpeg]

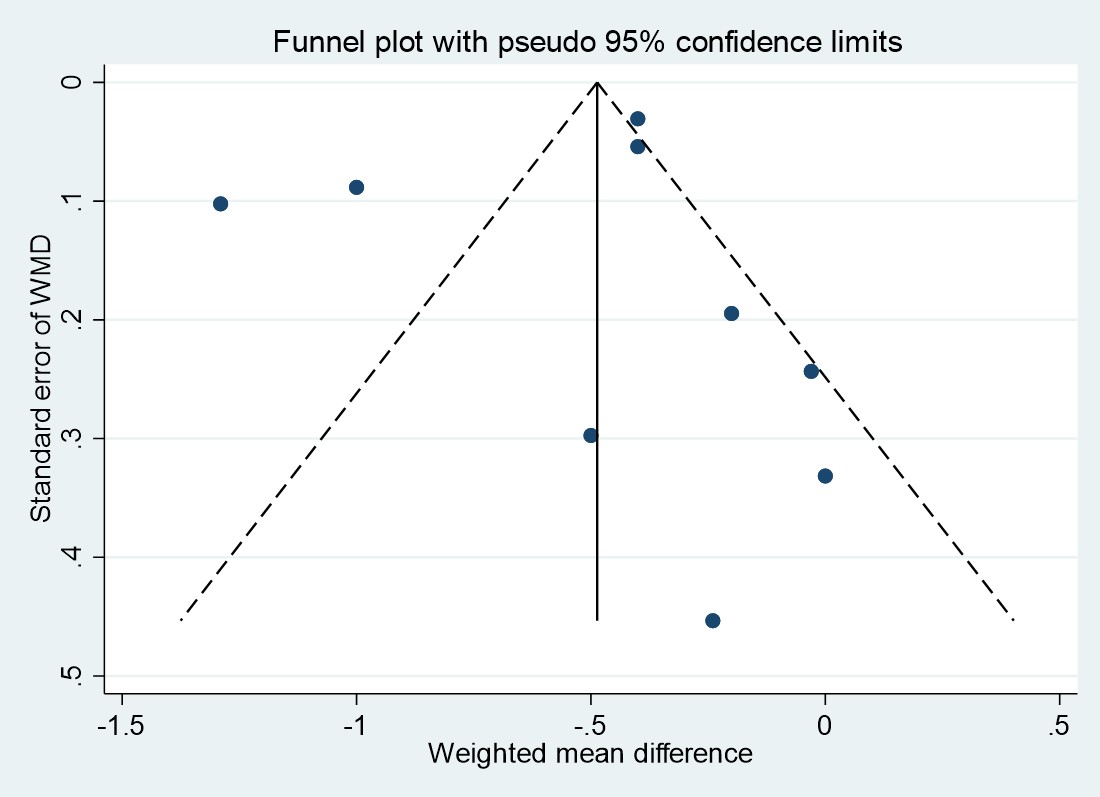

Supplement: Supplementary_figure_6_qfad026 [file supplementary_figure_6_qfad026.jpeg]

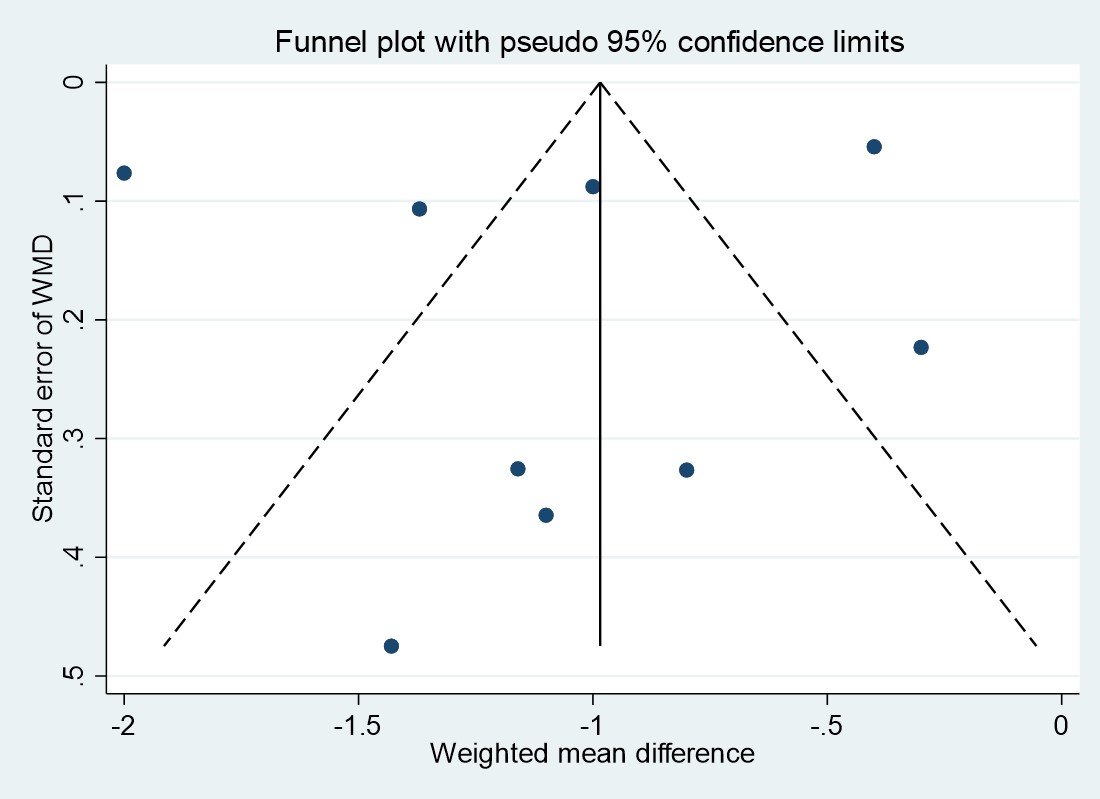

Supplement: Supplementary_figure_7_qfad026 [file supplementary_figure_7_qfad026.jpeg]
