# Supplementary material for: Impact of endometriosis on female sexual function: an updated systematic review and meta-analysis
Source: Sex Med. 2023 May 29;11(2):qfad026. doi: 10.1093/sexmed/qfad026 (PMC10226816; doi:10.1093/sexmed/qfad026)
Supplement: Supplementary_table_1_qfad026 [file supplementary_table_1_qfad026.docx]

**Supplementary table 1. Search strategy used to identify studies for inclusion in the meta-analysis**

| **PubMed** | **Embase** | **Scopus** |
| --- | --- | --- |
| #1 (endometriosis OR endometrioma OR abnormal uterine tissue OR adenomyosis OR uterine pathology)  #2 (sexual function OR libido OR infertility OR sexual function index OR sexual distress OR sexual arousal OR sexual desire OR orgasm OR lubrication)  #3 (#1 AND #2)  #4 (Addresses[ptyp] OR Autobiography[ptyp] OR Bibliography[ptyp] OR Biography[ptyp] OR pubmed books[filter] OR Case Reports[ptyp] OR Congresses[ptyp] OR Consensus Development Conference[ptyp] OR Directory[ptyp] OR Duplicate Publication[ptyp] OR Editorial[ptyp] OR Systematic reviews OR Meta analysis OR Festschrift[ptyp] OR Guideline[ptyp] OR In Vitro[ptyp] OR Interview[ptyp] OR Lectures [ptyp] OR Legal Cases[ptyp] OR News[ptyp] OR Newspaper Article[ptyp] OR Personal Narratives [ptyp] OR Portraits[ptyp] OR Retracted Publication[ ptyp] OR Twin Study[ptyp] OR Video-Audio Media[ptyp])  #5 (#3 NOT #4) | (endometriosis or abnormal uterine tissue or adenomyosis or uterine pathology or endometrioma and (sexual function) or (sex*) AND 'endometriosis':ti,ab,kw or “sexual function’; ti,ab,kw  or “sexual performance”: ti,ab,kw | #1 (endometriosis) or (adenomyosis) or (uterine pathology) OR TITLE-ABS-KEY (sexual dysfunc*)  #2 (sexual performance) or (sexual desire) or (pain) or (libido) OR TITLE-ABS-KEY (sexual function)  #1 and #2 |
