# Supplementary material for: Impact of endometriosis on female sexual function: an updated systematic review and meta-analysis
Source: Sex Med. 2023 May 29;11(2):qfad026. doi: 10.1093/sexmed/qfad026 (PMC10226816; doi:10.1093/sexmed/qfad026)
Supplement: Supplementary_table_3_qfad026 [file supplementary_table_3_qfad026.docx]

**Supplementary table 3. Author’s judgements about study quality using the adapted Ottawa-Newcastle Risk of Bias Assessment tool**

|  | Somigliana et al (2020) | Ashrafi et al (2022) | Kling et al (2022) | Tripoli et al (2011) | Ferrero et al (2005) | Giuliani et al (2016) |
| --- | --- | --- | --- | --- | --- | --- |
| Representativeness/appropriateness of participant selection  Random or consecutive recruitment=Y  Convenience sample=N  Not reported or unclear | Y | Y | Y | Y | Y | Y |
| Control for baseline differences in cohorts  Similarity of groups at baseline or adjustment in analyses=Y  No attempt to control or adjust=N  Not reported=NR | Y | Y | Y | Y | Y | Y |
| Loss to follow-up  Explanation provided for loss of participants and/or intention to treat=Y  No explanation =N | Y | Y | Y | Y | Y | N |
| Masking of exposure to outcomes assessor  Description of masking=Y  No masking or no description =N | Y | Y | Y | N | Y | Y |
| Ascertainment of condition  Description of ascertainment/diagnostic criteria=Y  No description or patient self-report=N | Y | Y | Y | Y | Y | Y |
| Documentation of other treatment modalities  Documentation=Y  No documentation=N | Y | N | Y | Y | N | Y |
| Extent to which valid outcomes are described  Adequate description of outcome=Y  Insufficient detail regarding outcome or follow-up time=N | Y | Y | Y | Y | Y | Y |
| Prespecification of harms, mode of harms collection  Description of a list of harms assessed or monitoring=Y  No such description or passive harms collection=N  No adverse events reported=NA | Y | N | Y | Y | N | Y |
| Financial Conflict of interest (COI)  Funding source reported=Y  Funding source not reported=N | Y | Y | Y | Y | Y | Y |
